# Reliability and validity of the Center for Epidemiologic Studies Depression Scale (CES-D) among suicide attempters and comparison residents in rural China

**DOI:** 10.1186/s12888-015-0458-1

**Published:** 2015-04-09

**Authors:** Li Yang, Cun-Xian Jia, Ping Qin

**Affiliations:** 1 Department of Epidemiology, Shandong University School of Public Health, Jinan, 250012 Shandong China; 2 Shandong University Center for Suicide Prevention Research, Jinan, 250012 Shandong China; 3 National Centre for Suicide Research and Prevention, Institute of Clinical Medicine, University of Oslo, Oslo, Norway

**Keywords:** Center for Epidemiologic Studies Depression Scale, Rural, Attempted suicide, Reliability, Validity

## Abstract

**Background:**

Depression is an important public health problem and is closely associated with suicidal behavior in the population. Although the Center for Epidemiologic Studies Depression Scale (CES-D) is widely used for assessment of depression, the psychometric characteristics of this scale have not been explored in studies of suicide attempters and local residents in rural areas.

**Methods:**

In this study, reliability and validity of CES-D were assessed in 409 suicide attempters and 409 comparison residents from rural China and through internal consistency analysis and confirmatory factor analysis (CFA).

**Results:**

Cronbach’s alpha values of the CES-D were 0.940 and 0.895 in, respectively, suicide attempters and comparison residents. CES-D scores were significantly correlated with the scores of Trait Anxiety Inventory (TAI) and Beck Hopelessness Scale (BHS) in both the suicide attempters and the comparison residents. Confirmatory factor analyses indicated that 3-factor structure (positive affect, interpersonal problems, depressive mood and somatic symptoms combined) with 14 items (excluding items 9, 10, 13, 15, 17, and 19) had the best fit in these two populations.

**Conclusions:**

The CES-D scale has satisfactory reliability and validity when used for assessing depression in suicide attempters and comparison residents in rural China.

## Background

Depression is an important public health problem and confers one of the most important risk factors for suicidal behavior in the general population regardless of sex and age groups [1,2]. Serious bouts of depression in individuals with a history of suicidal behavior could influence their capacity for normal life in the future [3,4]. Therefore, accurate diagnosis of depression in patients who attempted suicide is crucial for clinical treatment and for follow-up care of the patients [5,6]. The accuracy of depression assessment, however, highly relies on the validity of the tools that can be used for the assessment.

The Center for Epidemiologic Studies Depression Scale (CES-D), a self-report questionnaire, was developed to screen for depression, to assess depressive symptoms and to detect risks of having depressive disorder of a person [7]. The CES-D has shown to have generally good reliability and validity for depression assessment in various populations [8-10]. For instance, the CES-D was a useful tool in identifying clinical depression in Chinese American women and exhibited good construct validity and satisfactory internal consistency, albeit a cultural response bias was also detectable [11]. In spite of the popularity of the CES-D, there are areas of concern in terms of its factor structure and detail items [12-15]. Comparing with the original model, which is a four-factor model (depressed, somatic, interpersonal, and positive) and comprises 20 items [7], there have been various recommendations in studies validating this scale in specific populations. In a study of Chinese adolescents, the CES-D gave consistent results across the genders on the assessment of specific depressive symptom manifestations (i.e., depressed affect, positive affect, and somatic complaints) [13]. A few other studies from China have demonstrated that the four-factor model (depressed, somatic, interpersonal, and positive) fitted very well [14,15].

When applying the CES-D for research, it is important to examine whether the scale is reliable and valid for the study population, because, as indicated in the CES-D instruction, different ethnic and socio-demographic groups may have different factor structures [16]. Table 1 lists the original model and the factor models recommended in the recent literature. There have been obvious variations in the most suitable factor model when applying the CES-D in various populations. Aside from the original four-factor model, two-factor and three-factor models have also been recommended for specific groups of populations. In this study, we want to evaluate the psychometric characteristics of the CES-D in two sample populations comprising 409 suicide attempters and 409 paired comparison residents from rural China. We also want to examine which of the CES-D structure models recommended in the literature is most applicable to our study populations.

**Table 1:** **List of the original model and the recommended models on factor structures of the CES-D**

Reference	Factor (items)	CES-D item number and posited factor loading																			
		1	2	3	4	5	6	7	8	9	10	11	12	13	14	15	16	17	18	19	20
Radloff, 1977 Model C [7];	4 (20)	1	1	2	4	1	2	1	4	2	2	1	4	1	2	3	4	2	2	3	1
Shafer, 2006 [42];																					
Williams, 2007 [43]																					
Kohout, 1993 [44];	4 (19)	1	1	2	4	1	2	1	4	4		1	4	1	2	3	4	2	2	3	1
Carpenter, 1998 [45];																					
Irwin, 1999 Model A [46]																					
Schroevers, 2000 [47];	2 (20)	1	1	1	2	1	1	1	2	1	1	1	2	1	1	1	2	1	1	1	1
Rivera-Medina, 2010 [48]																					
Bush, 2004 Both Sexes [49]	4 (20)	1	3	1	4	1	1	1	4	2	1	1	4	3	2	2	4	3	2	2	1
Ying, 1988 [50];	3 (20)	1	1	1	2	1	1	1	2	1	1	1	2	1	1	3	2	1	1	3	1
Zhang, 2012 [8]																					
Carleton, 2013 [18]	3 (14)	1	1	2	3	1	2	1	3			1	3		2		3		2		1

## Method

### Study population

Six disease surveillance counties in Shandong Province (i.e., Jyu’nan, Lijin, Ningyang, Penglai, Tengzhou, and Zoucheng) were selected as study sites for data collection. Consecutive cases of rural residents aged 15–70 years old who attempted to kill themselves and therefore were sent for emergent treatment at one central general hospital of these Counties during the period from October 1, 2009 to March 31, 2011, were recruited as the cases of this study. The CDC (Centers for Disease Prevention and Control) of each County, as a routine, collected new incident cases from the hospital on a daily basis and provided us the information of the suicide attempters. In total, 1070 suicide attempters were reported during the study period. Of these individuals, 248 provided a made-up name or imprecise living address at the time of hospital treatment for suicide attempt, 369 were not at home during the follow-up surveys, and 44 refused to participate in the study. Therefore, 409 suicide attempters were finally included in this study, corresponding to a participation rate of 38.2%. There were no significant differences in the age (*t* = 1.088, *P* = 0.277) or gender (*χ*^2^ **=** 0.060, *P* = 0.807) of the interviewed cases versus those not interviewed.

In order to facilitate a comparison, 409 comparison residents were recruited into the study, on the basis that these individuals had no history of suicide attempts, and were 1:1 matched to the suicide attempters on gender, age (within 3 years) and village of residence.

### Procedure of data collection

The staff members of local CDCs were responsible for collecting information on suicide attempters treated in hospitals at the county level. Following the reported information from the CDC, interviews to the study cases were arranged with the help of the local CDC. The interviews were generally held one month after the attempted suicide in order to prevent undermining of the emotional stability of suicide attempters. Village doctors assisted the trained interviewers to find the homes of suicide attempters and comparison residents. Written informant consent was obtained from each subject prior to the interview. The interviews were undertaken face-to-face and tape-recorded upon the consent of the participants at the participants’ homes or the village clinics and without a third person present. Each interview lasted approximately 1.5 hours in duration.

### Instruments

Besides personal socio-demographic information such as gender, age, education level, marital status, occupation, and religious status, the following instruments were used for the data collection.

The CES-D comprises 20 items, and employs four-point Likert scales, ranging from “rarely or none of the time” (0 point) to “most or all of the times” (3 points). The total score ranges from 0 to 60, in which a higher score indicates more severe depressive symptoms [7]. Generally, a total CES-D score of 16 or greater can be considered indicative of depression [17]. But the validity and psychometric properties of several items (e.g., Items 7, 15,17, 19 ) on the CES-D have been questioned by the researchers [18].

The Trait Anxiety Inventory (TAI) of the State-Trait Anxiety Inventory (STAI) (Spielberger, 1983) consists of 20 statements and is usually used to evaluate respondents’ general tendency to perceive situations as threatening [19]. The total score on the TAI ranges from 20 to 80 [20]. In the present study, the Cronbach’s alpha values for the TAI in suicide attempters and comparison residents were 0.903 and 0.852, respectively.

The Beck Hopelessness Scale (BHS) [21,22] is a 20-item tool designed to measure three major aspects of hopelessness: feelings about the future, loss of motivation, and expectations. The BHS is a 5-point Likert scale, with answers from 1 (complete match) to 5 (in complete opposition), and a total score between 20 and 100. The Cronbach’s alpha values for the BHS were 0.954 and 0.883, respectively, for suicide attempters and comparison residents in this study.

### Data analysis

Data were analyzed via SPSS 16.0 (IBM SPSS, Inc. in Chicago, Illinois, USA) and Student version of LISREL 8.7 (Scientific Software International, Inc., Lincolnwood, IL, USA). A multivariate conditional logistic regression analysis was used to evaluate association between depression and attempted suicide. Reliability was assessed via assessment of internal consistency (Cronbach’s alpha). The scores of TAI and BHS were included in the analysis in order to evaluate the criterion validity of the CES-D via calculation of their correlation coefficients. Factor structures of the CES-D in the two study populations were examined through confirmatory factor analysis (CFA). Each model was evaluated by the following indices for fitness: 1) chi-square (values should not be significant); 2) chi-square/df ratio (values should be less than 5.0); 3) Comparative Fit Index (CFI) must be greater than 0.90; 4) the Standardized Root Mean Square Residual (SRMR) must be less than 0.10;5) Root Mean Square Error of Approximation (RMSEA) must be less than 0.08 with 90% confidence interval values below 0.10; and 6) lower values of Expected Cross-Validation Index (ECVI) indicate a closer fit across different models [18].

### Ethics statement

The study was approved by the Ethics Committee of Shandong University School of Public Health. All subjects signed the informed consent form. For subjects under 18 years of age, their parents also signed on the informed consent form.

## Results

### Demographic characteristics of the study samples

The population of 409 suicide attempters comprised 132 (32.3%) males and 277 (67.7%) females. The male to female ratio was 1:1.72. Because of the use of a paired case–control design, suicide attempters and comparison residents had virtually the same age and gender distribution.

As illustrated in Table 2, there were no significant differences in marital and religious status between the two study populations. However, suicide attempters were more often to be peasants and had a relatively lower level of education and higher scores of the CES-D as compared with the comparison residents (*P*s < 0.001). Therefore, the suicide attempters and the comparison residents could be regarded as different groups and should not be integrated into one sample when assessing the psychometric characteristics of the CES-D.

**Table 2:** **Characteristics of suicide attempters (N = 409) and comparison residents (N = 409), and the influences of these variables on suicide attempt**

Variables	Suicide attempters N (%)	Controls N (%)	Test of difference		Effect on suicide attempt		
			χ2	*P*	OR	95% CI	*P* *
Age			0.04	0.921	1.05	(0.92-1.20)	0.481
<60	349 (85.3)	351 (85.5)					
≥60	60 (14.7)	58 (14.2)					
Gender			<0.001	1	1.00	(0.74-1.34)	0.418
Female	277 (67.7)	277 (67.7)					
Male	132 (32.3)	132 (32.3)					
Education level			31.61	<0.001	2.02	(1.14-3.61)	0.017
≤8 years	383 (93.6)	329 (80.4)					
>8 years	26 (6.4)	80 (19.6)					
Marital status			1.54	0.256	0.92	(0.44-1.90)	0.816
Married	349 (85.3)	361 (88.3)					
Others	60 (14.7)	48 (11.7)					
Occupation			17.15	<0.001	1.75	(1.03-2.96)	0.034
Peasant	299 (73.1)	243 (59.4)					
Others	110 (26.9)	166 (40.6)					
Religious			0.37	0.685	4.37	(1.09-17.53)	0.038
No	395 (96.6)	398 (97.3)					
Yes	14 (3.4)	11 (2.7)					
Score of CES-D			270.23	<0.001	33.14	(15.21-72.20)	<0.001
≥16	247 (60.4)	24 (5.9)					
<16	162 (39.6)	385 (94.1)					

*P*: for comparison on the distribution of demographic characteristics between suicide attempters and controls.

P*: for multivariate logistic regression analysis.

OR: Odds ratio, derived from multiple logistic regression analysis.

Multivariate conditional logistic regression analysis was performed to assess the relative influence of the variables in the panel on risk for attempted suicide (Table 2). Depression was significantly associated with attempted suicide with an odds ratio (OR) of 33.140 (95% CI: 15.212-72.198) after the adjustment of the effects of other factors.

Comparing scores of men and women by *t*-tests indicated that there were not statistically significant differences by gender (*P* > 0.05) on most CES-D items except on CES-D items (17,19) in suicide attempters and CES-D items (4, 5, 10, 17, 18, 20) in comparison residents. Meanwhile, the sizes of gender effect were negligible (*r*^*2*^ < 0.01) for all items in both study populations.

### Internal consistency

Internal consistency was of an acceptable level for suicide attempters and comparison residents, with Cronbach’s coefficient alphas of 0.940 and 0.895, respectively, for the two populations. As depicted in Table 3, the item-correlation coefficient values were also significant (*Ps* < 0.01). Pearson coefficient values produced from an item-if-deleted analysis ranged from 0.298 to 0.839, and 0.259 to 0.728, respectively, for suicide attempters and comparison residents.

**Table 3:** **Internal consistency: sensitivity analysis for the CES-D in suicide attempters and comparison residents**

	Suicide attempters				Controls			
Items	M	SD	*r*	*α*	M	SD	*r*	*α*
Depression 1	22.21	4.04	0.74	0.94	2.92	2.50	0.47	0.89
Depression 2	22.54	4.05	0.66	0.94	2.87	2.49	0.40	0.89
Depression 3	22.25	4.04	0.75	0.94	2.94	2.48	0.68	0.88
Depression 4	22.16	4.05	0.65	0.94	2.85	2.47	0.50	0.89
Depression 5	22.51	4.05	0.67	0.94	2.93	2.52	0.26	0.89
Depression 6	21.93	4.03	0.84	0.94	2.87	2.46	0.70	0.88
Depression 7	22.33	4.04	0.73	0.94	2.83	2.46	0.59	0.88
Depression 8	22.06	4.04	0.72	0.94	2.88	2.48	0.54	0.89
Depression 9	22.31	4.04	0.74	0.94	2.97	2.50	0.52	0.89
Depression 10	23.26	4.13	0.30	0.95	3.00	2.52	0.36	0.89
Depression 11	22.35	4.05	0.65	0.94	2.75	2.46	0.51	0.89
Depression 12	21.81	4.04	0.74	0.94	2.88	2.47	0.57	0.88
Depression 13	22.37	4.05	0.68	0.94	2.91	2.50	0.43	0.89
Depression 14	22.67	4.07	0.59	0.94	2.93	2.49	0.56	0.89
Depression 15	23.23	4.13	0.31	0.95	2.96	2.50	0.44	0.89
Depression 16	22.01	4.04	0.72	0.94	2.89	2.48	0.49	0.89
Depression 17	22.81	4.09	0.56	0.94	3.01	2.52	0.47	0.89
Depression 18	21.98	4.03	0.78	0.94	2.85	2.45	0.71	0.88
Depression 19	23.26	4.13	0.30	0.95	3.01	2.52	0.43	0.89
Depression 20	22.18	4.03	0.80	0.94	2.88	2.45	0.73	0.88

M: Scale mean if item deleted; SD: Standard deviation; *r*: Corrected item-total correlation.

α: Cronbach’s alpha if item deleted.

### Criterion validity

Mean values of CES-D, BHS, and TAI scores were, respectively, 23.59 (SD = 1.69), 57.49 (SD = 2.07), and 38.59 (SD = 1.03) for suicide attempters, and respectively, 3.04 (SD = 0.63), 32.46 (SD = 0.99), and 30.47 (SD = 0.69) for the comparison residents.

CES-D scores were significantly correlated with BHS scores in both suicide attempters (*r* = 0.72) and comparison residents (*r* = 0.44) (*Ps* < 0.001). Similarly, CES-D scores were also significantly correlated with TAI scores in suicide attempters (*r* = 0.46) and comparison residents (*r* = 0.58) (*Ps* < 0.001).

### Factor structure of the CES-D

The original model and 5 currently recommended models were assessed in the sample populations of suicide attempters and comparison residents using the confirmatory factor analysis (CFA). Detailed results are listed in Table 4. The results showed that the ECVI of both groups were lowest in the 3-factor model comprising 14-item derived by Carleton and his colleagues [18]. This means that this model has the best fit for the factorial structure in the two study populations. In suicide attempters, χ^*2*^/*df* was less than 5. The RMSEA was less than 0.10. The CFI was over 0.90, and the SRMR was less than 0.05. In the sample of comparison residents, χ^*2*^/*df* was close to 5. The RMSEA was close to 0.10. The CFI was over 0.90, and the SRMR was close to 0.05.

**Table 4:** **The fit indices of the existing models on factorial structures, derived from confirmatory factor analyses (CFA)**

	x2	df	x2/df	CFI	SRMR	RMSEA (90% CI)	ECVI (90% CI)
Radloff, 1977 [7]; Model C; Shafer, 2006 [20]; Williams, 2007 [43]							
Suicide attempters	617.7	145	4.26	0.97	0.056	0.088 (0.080,0.095)	1.69 (1.52,1.89)
Controls	907.01	164	5.53	0.91	0.078	0.110 (0.100,0.120)	2.56 (2.33,2.81)
Kohout, 1993 [44]; Carpenter, 1998 [45]; Irwin 1999 Model A [46]							
Suicide attempters	494.11	146	3.38	0.98	0.044	0.077 (0.070,0.084)	1.44 (1.29,1.62)
Controls	668.22	129	5.18	0.92	0.069	0.100 (0.092,0.110)	1.81 (1.62,2.02)
Schroevers, 2000 [47]; Rivera-Medina, 2010 [48]							
Suicide attempters	665.58	151	4.41	0.96	0.060	0.092 (0.085,0.099)	1.85 (1.66,2.06)
Controls	1102.64	169	6.52	0.88	0.085	0.120 (0.110,0.130)	3.12 (2.86,3.39)
Bush, 2004 both sexes [49]							
Suicide attempters	695.37	164	4.24	0.97	0.056	0.088 (0.081,0.095)	1.90 (1.71,2.10)
Controls	1056.47	164	6.44	0.89	0.083	0.140 (0.110,0.130)	2.95 (2.70,3.22)
Carleton, 2013 [18]							
Suicide attempters	241.36	74	3.26	0.98	0.036	0.076 (0.066,0.087)	0.77 (0.66,0.89)
Controls	413.64	74	5.58	0.93	0.070	0.110 (0.097,0.087)	1.18 (1.03,1.35)
Ying, 1988 [50]; Zhang, 2012 [30]							
Suicide attempters	546.59	167	3.27	0.98	0.044	0.076 (0.069,0.083)	1.60 (1.43.1.78)
Controls	977.73	167	5.85	0.90	0.080	0.110 (0.110,0.120)	2.82 (2.57,3.08)

## Discussion

This study sought to assess the reliability and validity of the CES-D in the assessment of depression in Chinese rural suicide attempters and community comparison residents. The findings indicate that the CES-D has satisfactory reliability in depression assessment situated within the Chinese culture and that the three-factor model with 14 items of the CES-D [18] has an acceptable goodness of fit in the two sample populations of suicide attempters and comparison residents in rural China. Such results were consistent with previous validation of this scale in the populations of suicide completers and comparison residents from rural China [8,17].

The CES-D has exhibited a satisfactory reliability in a number of studies of the general population, with a high Cronbach’s alpha value, for instance, in Armenian (0.89 for women and 0.83 for men), Dutch (0.93), and English and the Spanish people (0.91 and 0.92 respectively) [23-25]. The CES-D has also showed a satisfactory reliability in specific groups of populations. For example, a Canadian study reported a Cronbach’s alpha of 0.88 for the overall CES-D in patients with systemic sclerosis [26]. The Cronbach’s alpha was also high (0.84) in a sample of Dutch elderly [27]. In this study, the Cronbach’s alpha was above 0.80 for both suicide attempters and comparison residents, indicating a good internal consistency and a high reliability of the CES-D when used in these populations.

The present study shows that CES-D scores were significantly higher in suicide attempters than that in comparison residents, and that depression was significantly associated with attempted suicide. These results are highly in line with the literature that depression is an important risk factor for suicidal behavior [28,29]. Such findings underscore the need and importance of depression assessment in suicide prevention practices.

In this study, the TAI and BHS were used to evaluate the criterion validity of the CES-D. The TAI is designed to assess trait anxiety personality, which embodies stable individual differences in tendency toward anxiety and general proneness to respond with anxiety to perceived threats in the environment [19]. This inventory has adequate psychometrics for measuring trait anxiety in suicide victims and living controls in rural China [30]. Although anxiety is not a necessary syndrome of pre-suicide, more than 70% of suicide attempters have an anxiety disorder [31] and the disorder shares some clinical characteristics with depression and confers an important risk factor for suicide attempt and completion [32]. On the basis of the cognitive theory of depression, hopelessness increases the risk of depression, because it gives rise to negative feelings including worthlessness, loss, and expected failure in response to a stressor [33]. Previous studies have shown that, the BHS has a high degree of internal consistency and validity in the context of Chinese culture [34-36] and that hopelessness is strongly related to suicide attempt [11,37]. In this study, the CES-D scores were significantly and positively correlated with the scores of both TAI and BHS, which indicate that the CES-D has good criterion validity in the two study populations.

In utilization of the CES-D theory-driven confirmatory analyses may benefit more than do exploratory analyses. The present study is the first one that used the same study populations to examine the fit indices of the CES-D factor structure of the original model and the models recommended in previous studies. The factor structure proposed by Carleton et al. [18] produced the best fit indices in our study populations and was consistent with current DSM IV-TR conceptualization of depression. This factor structure eliminates the items of 9, 10, 13, 15, 17, and 19, and includes 3 structural factors of negative affect (items 3, 6, 14, 18), anhedonia (items 4, 8, 12, 16), and somatic complaints (items 1, 2, 5, 7, 11, 20).

It is not easy to explain why the three-factor model with 14 items proposed by Carleton et al. [18] shows the best fit in our study populations. Several studies have questioned the validity and psychometric properties of the scale items of 15, 17 and 19 [18] as well as the items of 9, 10 and 13 [7]. It is also possible that variations of socio-demographic characteristics of study populations as well as culture differences contribute to the observed differences of results from the present study to other studies in the literature. For instance, in stroke patients in Korea [38], the 5-factor structure was supported (loss of vitality, positive affect, psychomotor retardation, negative affect, and interpersonal problems) and could explain 61.25% of the variance. In a Hong Kong study of Chinese married couples [39], only two factors were derived (depressive symptom factor and interpersonal problem factor). In contrast to the depressed English-speaking smokers, a different pattern of three factors has emerged in the depressed Spanish -speaking smokers [25]. Zhang et al. proposed a three-factor model with 20 items in the samples of suicide informants and controls in rural China [8]. In Chinese adolescents, 3 factors explaining 48.58% of the total variance were produced to cover “depressed affect”, “somatic complaints”, and “positive affect” [32]. The slight difference of depression structure in suicide attempters from the structure in the comparison residents might to some extent be induced by differences in education level, occupation and scores of CES-D between the two populations. Further studies with larger samples are certainly needed for confirmation of the observations from the present study.

Some shortcomings of this study should be noted. The bias of most concern in this study is the recall bias because the data were collected through interview. The use of the TAI and the BHS to assess the criterion validity of the CES-D may not be as ideal as the use of, for instance, the HAMD [40], and Zung Self-Rating Depression Scale [41]. Other limitations include not using diagnostic interview, the absence of behavioral correlates, and an absent experimental design, relative small populations of study, etc.

## Conclusions

The current study provides preliminary evidence on the reliability and validity of the CES-D in suicide attempters and comparison residents from rural China. It is the first study examining the fit indices of all suggested factor structure models of this scale in the two study populations simultaneously. The results indicate that the CES-D has satisfactory reliability and validity for the assessment of depression or depressive symptoms in suicide attempters and comparison residents in rural areas of China.
